# Supplementary material for: Splice-Junction-Based Mapping of Alternative Isoforms in the Human Proteome
Source: Cell Rep. Author manuscript; Available in PMC 2020 Jan 15. (PMC6961840; doi:10.1016/j.celrep.2019.11.026)
Supplement: 3 [file NIHMS1546469-supplement-3.zip › DF2/PXD009737/TestisTrypsin-194-Q9HBL0-LSALGLKPHNPADILLHPTGEPR.pdf]

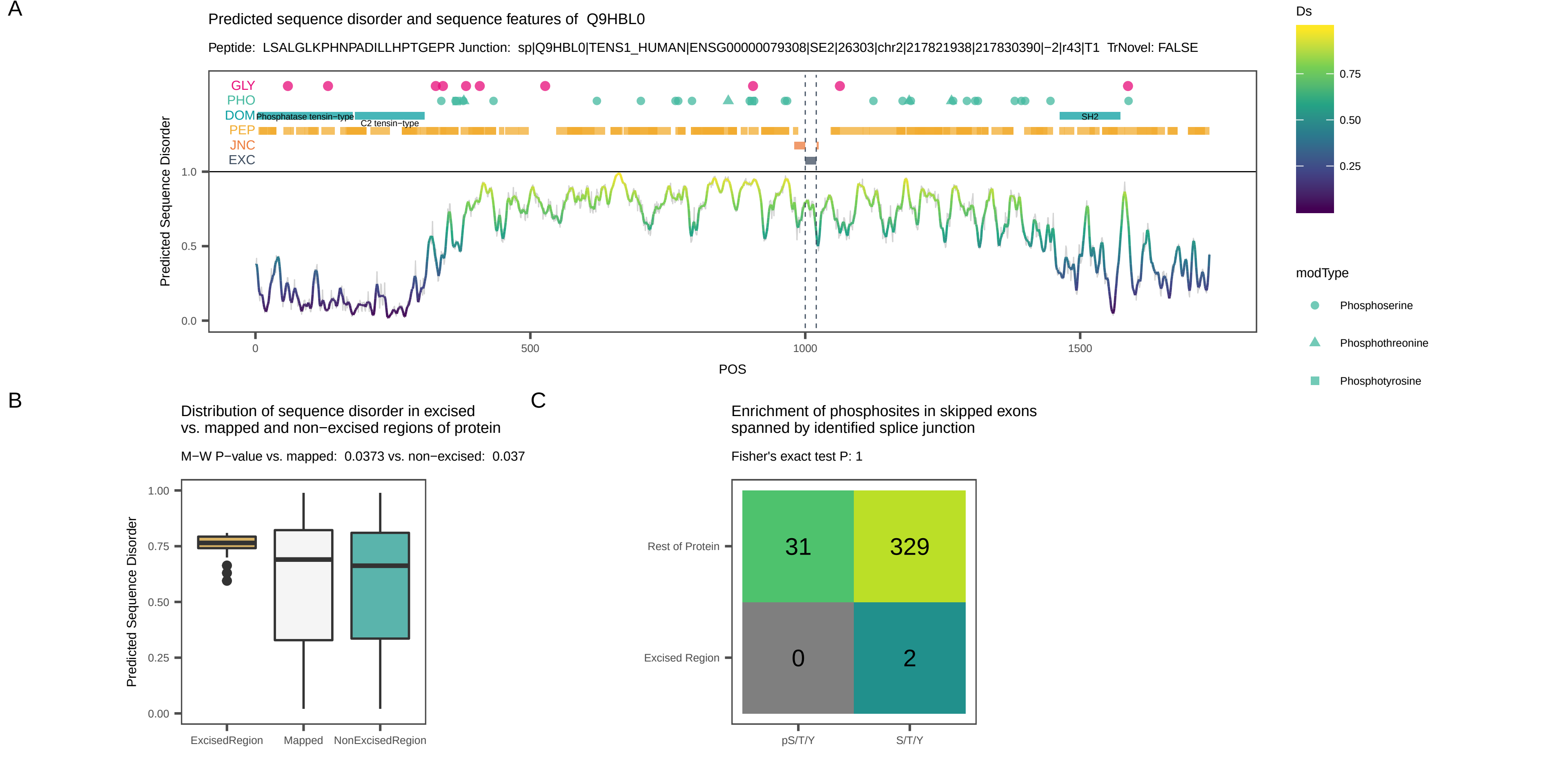

A
Predicted sequence disorder and sequence features of Q9HBL0
Peptide: LSALGLKPHNPADILLHPTGEPR Junction: sp|Q9HBL0|TENS1_HUMAN|ENSG00000079308|SE2|26303|chr2|217821938|217830390|−2|r43|T1 TrNovel: FALSE
GLY
PHO
DOM
Phosphatase tensin−type
C2 tensin−type
SH2
PEP
JNC
EXC
Predicted Sequence Disorder
1.0
0.5
0.0
0
500
1000
1500
POS
Ds
0.75
0.50
0.25
modType
Phosphoserine
Phosphothreonine
Phosphotyrosine
B
Distribution of sequence disorder in excised
vs. mapped and non−excised regions of protein
M−W P−value vs. mapped: 0.0373 vs. non−excised: 0.037
Predicted Sequence Disorder
1.00
0.75
0.50
0.25
0.00
ExcisedRegion
Mapped
NonExcisedRegion
C
Enrichment of phosphosites in skipped exons
spanned by identified splice junction
Fisher's exact test P: 1
Rest of Protein
Excised Region
31
329
0
2
pS/T/Y
S/T/Y
